# Single‐Cell Insights Into Macrophage Subtypes in Pulmonary Infections

**DOI:** 10.1002/advs.202510758

**Published:** 2025-11-21

**Authors:** Zhaoheng Lin, Yuxiao Zheng, Yu Zhong, Hongyan Wang

**Affiliations:** ^1^ Key Laboratory of Systems Health Science of Zhejiang Province School of Life Science Hangzhou Institute for Advanced Study University of Chinese Academy of Sciences Hangzhou 310024 China; ^2^ Key Laboratory of RNA Science and Engineering & State Key Laboratory of Cell Biology CAS Center for Excellence in Molecular Cell Science Shanghai Institute of Biochemistry and Cell Biology University of Chinese Academy of Sciences Chinese Academy of Sciences Shanghai 200031 China; ^3^ Shanghai Cancer Institute, Ren Ji Hospital School of Medicine, Shanghai Jiao Tong University Shanghai 200240 China

**Keywords:** cell fate plasticity, macrophage subtypes, pulmonary infections, single‐cell omics

## Abstract

Macrophages are pivotal innate immune cells that play essential roles in pathogen recognition, inflammation modulation, and tissue repair during pulmonary infections. Macrophages have remarkable plasticity that is shaped by diverse external stimuli to adapt to the dynamic lung microenvironment. Traditional models of macrophage polarization (M1/M2) cannot capture the full complexity of macrophage heterogeneity and diverse functions during lung infections. Recent advances in single‐cell omics have provided new insights into distinct macrophage subtypes, revealing their unique transcriptional profiles across various stages of infection. This review focuses on the functional plasticity of pulmonary macrophages and how environmental cues modulate their activation and effector functions. An integrative classification framework that defines six major functional macrophage subtypes in pulmonary infections, based on single‐cell omics with functional perspectives is proposed. This framework refines the understanding of macrophage heterogeneity and offers a foundation for developing targeted immunotherapeutic strategies against lung infections.

## Graphical abstract

This review highlights the dynamic plasticity of macrophages during pulmonary infections and proposes an integrative framework defining six functional subtypes: Inflam‐Ms, Hub‐Ms, Reg‐Ms, Prolif‐Ms, Memory‐Ms, and Senesc‐Ms. Single‐cell omics delineate their distinct roles in homeostasis and infection, refining our understanding of macrophage heterogeneity and its implications for infection biology and therapeutic exploration.

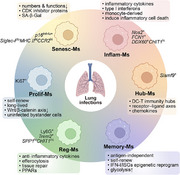

## 1 Introduction

Macrophages are important innate immune cells due to their remarkable functional diversity and large numbers at infection sites during host defense against invading pathogens.^[^ ^1^, ^2^, ^3^ ^]^ The unique anatomical and physiological features of the lung, including its extensive surface area and constant exposure to environmental stimuli, significantly influence macrophage functions.^[^ ^4^, ^5^ ^]^ Macrophages can detect dynamic microenvironment stimuli to modulate their own function as well as their interactions with various cell types, including adaptive immune cells and epithelial cells.^[^ ^6^ ^]^

Genetic fate mapping studies have revealed that lung macrophages originate from distinct lineage sources, including embryonically‐derived resident macrophages and monocyte‐derived macrophages recruited from the circulation during infection or inflammation.^[^ ^6^, ^7^, ^8^ ^]^ The lung harbors two major macrophage populations, alveolar macrophages (AMs) and interstitial macrophages (IMs), each with unique anatomical localization and functional specialization. AMs are the predominant resident subset, which primarily arises from embryonic precursors, including yolk sac macrophages and fetal liver monocytes, and establishes a long‐lived, self‐renewing population throughout life.^[^ ^7^, ^9^ ^]^ Under inflammatory or infectious conditions, AMs can be replenished by circulating monocytes. In contrast, IMs reside within the bronchial interstitium and are derived predominantly from blood monocytes.^[^ ^10^, ^11^, ^12^ ^]^ IMs highly express monocyte‐associated genes (e.g., *Cd14*, *Cd163*, *Csfr1*), supporting their monocytic origin.^[^ ^10^ ^]^ Both AMs and IMs coordinate immune surveillance, inflammation initiation and resolution, and tissue homeostasis. This functional synergy underscores their critical roles in lung immunity.^[^ ^13^, ^14^ ^]^ Understanding the functional plasticity of these macrophage populations is crucial for manipulating macrophage cell fates to increase the efficacy of pathogen clearance and tissue repair during pulmonary infections.

**Figure 1:**
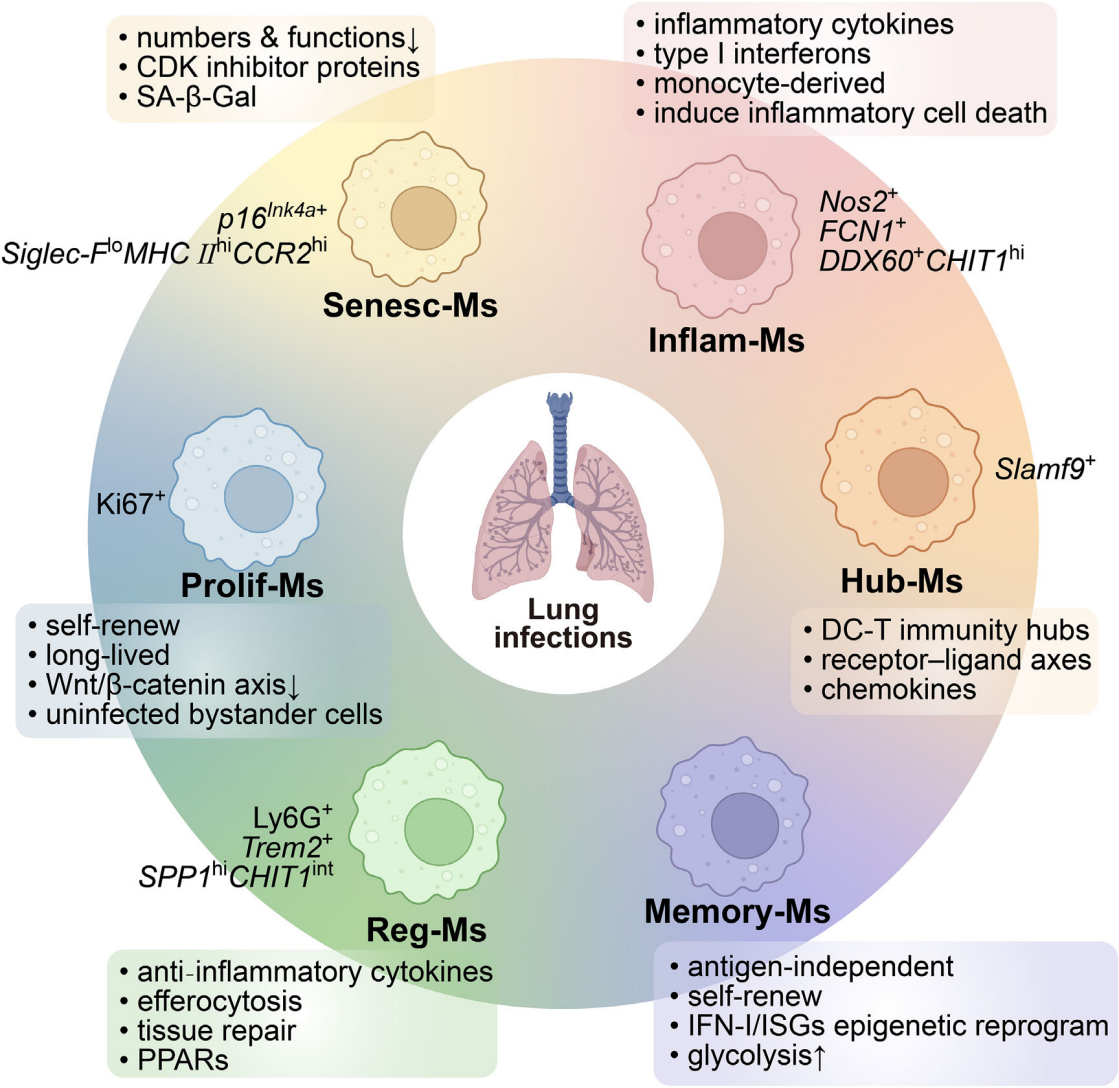
Characteristics of macrophage subtypes in pulmonary infections. Inflam‐Ms, inflammatory cytokine‐enriched macrophages; Hub‐Ms, immunity hubs‐associated macrophages; Reg‐Ms, immune regulatory macrophages; Prolif‐Ms, proliferating macrophages; Memory‐Ms, Memory macrophages; Senesc‐Ms, Senescent macrophages; PPARs, peroxisome proliferator‐activated receptors. Created with BioRender.com.

The proinflammatory (M1)/anti‐inflammatory (M2) macrophage polarization paradigm cannot capture the full spectrum of macrophage heterogeneity, because of its limitation including only reflecting in vitro polarization states and no cleanly mapping the in vivo phenotypes. To address these limitations, single‐cell omics have provided unprecedented resolution to explore macrophage heterogeneity, which could reveal previously unrecognized macrophage subpopulations, offering deeper insights into their functional states and transcriptional landscapes.^[^ ^14^, ^15^, ^16^, ^17^ ^]^ During pulmonary infections, the lung microenvironment is characterized by inflammation, hypoxia, and tissue damage, which can dynamically shape the functional state of macrophages.^[^ ^6^, ^18^ ^]^ This review discusses the dynamic plasticity of macrophage subtypes throughout pulmonary infections, delineates their distinct roles, and classifies them into six key subtypes: inflammatory cytokine‐enriched macrophages (Inflam‐Ms), immunity hubs‐associated macrophages (Hub‐Ms), immune regulatory macrophages (Reg‐Ms), proliferating macrophages (Prolif‐Ms), memory macrophages (Memory‐Ms), and senescent macrophages (Senesc‐Ms) (**Figure** **1**; **Table** **1**).

**Table 1:** Characterization and functional roles of macrophage subsets in lung infection.

Inflammatory cytokine‐enriched macrophages	
Human‐derived signature ^[^ ^107^, ^108^, ^117^, ^196^, ^197^, ^198^ ^]^: ***FCN1*** *, MAFF, KLF4, NFE2L2, BACH1, TOP1*, ***ISG15/20*** *, Lgals3, Ndufa4, Fabp5*, ***S100A8/9/10/12***, ***GBP1/5*** *, CD63, Fcer1g, HSPA6/A1A/A1B/B1/H1, IER3, LITAF, CASP1, ELK3, SERPINB1/9, UBE2B, BID, HNRNPH2, STARD4, KPNA4, FCGR3A, ELOVL5, LYZ, APOE, C1QA, B2M, VCAN, CORO1A, SELL, CFP, RNASE2, FPR1, COTL1, MPEG1, STAB1, MS4A6A, DNAJB1, BAG3, TIMP1, DEFB1, VAMP5, NINJ1, SOD2, BCL2A1, TYMP, SGK1, CTSB, ANKRD22, CALHM6, APOBEC3A, IDO1, TNFSF10, CLU, PPARG, TCF7L2, INHBA, SLC19A3, ITGB8, CD5L, IL17RB, SLC25A37, PLEK, GCH1, PLA2G7, SLAMF7, CYP1B1, CTSL, IFI6*, ***CXCL*** *5/10/* ***11*** */18*, ***CCL*** *2/* ***3*** */4/8/18/* ***20***, ***IL1B*** Mouse‐derived signature ^[^ ^48^, ^117^, ^199^, ^200^, ^201^ ^]^: ***S100a8/a9*** *, Sdc1, Cst7, Fam162a, Orm1, Clmp, Slc7a2, Saa3*, ***Nos2*** *, Adgre1, Clec4e, Ubd, Fcgr1/2b, Apoe, C1qa, Itgam, Ccr2, Ccr5*, ***Il1b, Tnfa*** *, Tnfsf10, Cxclc2, Cxcl3,Csf1,Il4, Myd88, Mafb, Irg1, Aif1, Hif1a*, ***Ccl*** *2/4/* ***5***, ***Irf1*** *, Irf7, Isg15, Ifitm3, Cxcl10*; CD68^hi^, CD40^hi^, CD38^+^, CD86^+^, iNOS^+^	
Induction type	Human: *Mycobacterium tuberculosis* (*Mtb*), *Mycobacterium bovis*, Bacillus Calmette‐Guérin (BCG), SARS‐CoV‐2 Mouse: *Klebsiella pneumoniae*, *Mtb*, Influenza A virus (IAV)
Function/Enriched pathway	Human: IFN response, Viral replication, Cytokine secretion, Neutrophil activation Mouse: IFN response, TNF signaling, Antigen processing and presentation, Cytokines and chemokines enrichment, NF‐κB signaling, Complement activation, Leukocyte migration, Glycolysis, Endocytosis
Immunity hubs‐associated macrophages	
Human‐derived signature ^[^ ^23^, ^107^ ^]^: *IFIT2, IFIT3, ISG15, AGRP, IGFBP2, LPL, PARAL1, CD52, PTPMT1, CYSLTR1, STMN1, MGST3, TSPO, ASP5, S100A4/13*, ***CXCL*** *8/9/* ***10*** */11*, ***CCL*** *7/* ***8*** */13/23/24* Mouse/hamster‐derived signature ^[^ ^14^, ^35^, ^117^, ^129^, ^130^ ^]^: ***Slamf9***, ***Spp1*** *, Siglec1, H2‐M2, Ubd, Cd38, Isg12, Cd80, Ctsd, Apoe, Lyz2, Fth1, Gdf15, Ccr2, Ccl5, Ccl6, Cxcl4/9/10/11/16, Il18, Il10, Il1a*, ***Il1b***, ***Tgfb1***	
Induction type	Human: SARS‐CoV‐2, *Mtb* Mouse/hamster: SARS‐CoV‐2, IAV, *Pseudomonas aeruginosa*
Function/Enriched pathway	Human: Monocytes and T cells recruitment, IFN response, Antigen processing and presentation, Chemokine‐mediated signaling Mouse/hamster: Neutrophil interaction, Lymphocyte interaction, Antigen processing and presentation, Chemokine‐mediated signaling, Lysosome, Peroxisome, TLR signaling
Immune regulatory macrophages	
Human‐derived signature ^[^ ^14^, ^107^, ^108^, ^198^, ^200^, ^202^, ^203^, ^204^, ^205^ ^]^: ***CD163***, ***CD206***, ***SPP1*** *, RHOA, HLA‐A, AZIN1, TUBA1A, FAM49B, NLN, RIPK2, HSPD1/90B1/H1, CD84, CD86, CD180, TAF7, DUSP3, FCGR2A, RIT1, PTP4A1, GNPGAB, DYSF, LGMN, RGS1, RNASE1, HMOX1, C1QC, SDS, ARL4C, CTSL, CTSZ, GPR183, NR1H3, FPR3, ABCA1, TTYH3, CREG1, A2M, NRP2, PLTP, GPNMB, LHFPL2, SMPDL3A, PLD3, MARCKS, SDC3, TMEM2* ***/*** *176A/176B, PMP22, MMP14, MS4A4A, CREM, APOE, IL7R, LIPA*, ***IL4***, ***IL13***, ***IL10*** *, CCL18, CCL13*, ***TGFB1*** Mouse/hamster‐derived signature ^[^ ^14^, ^48^, ^117^, ^141^, ^201^, ^206^ ^]^: *Csf1, Slamf7*, ***Arg1***, ***Mrc1***, ***Trem2***, ***Spp1***, ***C1q*** *a/b/c, Marco, Ctsb, Fcgr2b, Ifit1, Usp18, Irgm1, Irg1, Ptgs2, Ptafr, lcn2, Socs1/3, Msr1, Mmp14, Ccr1/2/7, Clec4b, Parp14, Il1rn*, ***Fizz1***, ***Cd163*** *, Slc7a2, Ear2, Malat1, Kcnq1ot1*, ***Apoe*** *, Aif1, Ms4a7, Mrc1*, ***Fn1*** *, Vim, Crip1, Pdgfc, Plet1, Cxcl12/16*, ***Il4/10/13***, ***Tgfb1*** *, Pparg, Klf4, Myc31, Rxra33*; Ly6G^+^, CD64^+^, Siglec‐F^lo^, CD11c^lo^, CD163^+^, CXCR4^hi^, MHC II ^hi^, CD101^hi^, CD319^hi^, CD177^lo^, CD206^hi^, ARG1^+^	
Induction type	Human: *Mtb*, IAV, SARS‐CoV‐2 Mouse/hamster: IAV, SARS‐CoV‐2, *Klebsiella pneumoniae*, *P. aeruginosa*, *Mtb*, *Nippostrongylus brasiliensis*
Function/Enriched pathway	Human: Neutrophil degranulation, Leukocyte differentiation, Regulation of plasma lipoprotein particle levels, Oxidative stress response, Lipid catabolic process, ECM remodeling Mouse/hamster: Oxidative phosphorylation, PPAR signaling, Proteasome, Calcium signaling, Wnt signaling, Cell‐substrate adhesion, Cell migration, Cell development, Protein kinase activity, Fatty acid oxidation
Proliferating macrophages	
Human‐derived signature ^[^ ^107^, ^117^ ^]^: *TYMS, STMN1*, ***TOP2A*** *, PCLAF*, ***MKi67*** Mouse/hamster‐derived signature ^[^ ^14^, ^117^, ^130^, ^152^, ^206^, ^207^ ^]^: ***Mki67*** *, Fbp1, Mx1, Irg1, Ifi44l, Lgals9, Unc93b1, Irf7, Isg15, Bst2, Ifit2/3, Bax, Apobec1, parp9, Rsad2, Rtp4, Adar, Clec4e, Nmi, Ager, Eif2ak2, Stmn1*, ***Top2a*** *, Il1r2, Saa3, Mcm3/4/5/6, Tff2*, ***c‐Myc*** *, Ccl2*	
Induction type	Human: BCG, *Mtb* Mouse/hamster: IAV, SARS‐CoV‐2, *P. aeruginosa*, *Mtb*
Function/Enriched pathway	Human: Proliferation Mouse/hamster: Proliferation, Chemokine signaling, NLR signaling, ECM receptor interaction, Ubiquitin mediated proteolysis, p53 signaling, MAPK signaling, Wnt signaling
Memory macrophages	
Mouse‐derived signature ^[^ ^3^, ^169^ ^]^: CD64, TLR2/4, CD80, CD86*, Cd36, Mip2, Cxcl1*; **MHC II^hi^**, Siglec‐F^hi^	
Induction type	Mouse: Adenoviruses, SARS‐CoV‐2, IAV
Function/Enriched pathway	Mouse: Antiviral immune response and IFN‐I signaling
Senescent macrophages	
Mouse‐derived signature ^[^ ^179^, ^181^, ^184^, ^189^, ^208^, ^209^, ^210^, ^211^, ^212^ ^]^: *Cd24a, Marco, Ctsd, Cybb, Pdpn, Cxcr1, Cxcr2, Pdk4, Slpi, S100a1, Plpp3, Gm12840, Cd63, Wfdc21, Serpine1, Car4, Gstm1, Wfdc17, Chil3, Spp1, Gpnmb*, ***Mfge8***, ***Cebpb*** *, Cxcl3, Ccl6*, ***Ccl2*** *, Il1b, Il12*, ***Il10*** *, Tnfa*, ***Ifnb***, ***Mif***, ***Cdkn2a***, ***Phlda3***, ***cpeb1*** *, Cxcl2, Mmp14, Ax1, Itgav, Chil1, Cd74, Cdkn2a, Ptgs1*; **MHC II^hi^**, CD11c^+^, CD11b^+^, Siglec‐F^lo^, CCR2^hi^, p16^Ink4a+^, **β‐galactosidase**	
Induction type	Mouse: *Mtb*, IAV, *Streptococcus pneumoniae*
Function/Enriched pathway	Mouse: Cell‐cycle arrest, Cellular senescence, Cell junction, Cell adhesion, Cell migration, PGE2‐EP2 signaling, Endocytosis

*Italic font* denotes signature genes of the macrophage clusters. The Roman font represents the protein markers of the macrophage clusters. The key genes reported in multiple studies are highlighted in **bold**.

## 2 Macrophage Plasticity in the Infected Pulmonary Microenvironment: From Homeostasis to Inflammation and Resolution

### 2.1 Overview of the Pulmonary Microenvironment and Macrophage Regulation

The pulmonary microenvironment contains various cells, such as alveolar epithelial cells, fibroblasts, endothelial cells, immune cells, and the lung microbiome, which cooperatively maintain lung homeostasis.^[^ ^2^, ^13^, ^19^ ^]^ Oxygen abundance, pH, the extracellular matrix (ECM) composition, and surfactants are key factors in the lung that modulate the functions of immune cells, particularly macrophages.^[^ ^20^, ^21^ ^]^ Additionally, the lung microbiome, including both pathogenic and symbiotic microorganisms, is crucial for maintaining the microecosystem balance. It enhances macrophage antimicrobial activity through reactive oxygen species (ROS) production and induces a spectrum of cytokines, including IL‐6, IL‐10, IL‐17, and TNF‐α.^[^ ^19^, ^22^ ^]^ When encountering pathogens, sensing host‐derived cues, or interacting with other cells in the lung microenvironment, macrophages activate distinct signaling pathways to dynamically adjust their phenotype and immune responses to meet the specific demands of the lung tissue.^[^ ^23^ ^]^ For example, pathogen‐associated molecular patterns (PAMPs) in pathogens can be recognized by pattern recognition receptors (PRRs) on the tissue‐resident AMs, such as Toll‐like receptors (TLRs),^[^ ^24^, ^25^ ^]^ and trigger a cascade of proinflammatory signaling pathways, driving macrophages into an inflammatory state.^[^ ^26^, ^27^ ^]^ Cytokines and chemokines in the lung further regulate macrophage proliferation and differentiation, and recruit additional immune cells to the site of infection, thereby enhancing pathogen clearance.^[^ ^6^ ^]^

Pulmonary macrophages are uniquely adapted to the lung environment, which is defined by direct air exposure, gas exchange, and constant contact with inhaled particles. Unlike macrophages in the liver, kidney, spleen, or heart, AMs and alveolar‐associated IMs predominantly sustain themselves through local self‐proliferation under homeostatic conditions.^[^ ^9^ ^]^ Circulating monocyte recruitment becomes prominent only during infection or injury.^[^ ^28^ ^]^ AMs, as resident macrophages directly guarding the gas‐exchange surface, must balance efficient pathogen clearance with the need to prevent excessive inflammation. Accordingly, AMs could remove pathogens and particulates and promote tissue recovery.^[^ ^29^ ^]^ This dual functionality contrasts with the tolerance‐oriented phenotype of intestinal macrophages toward commensals, the fibrosis‐promoting potential of liver macrophages, and the comparatively limited reparative role of kidney macrophages.^[^ ^30^, ^31^ ^]^ Morphologically, pulmonary macrophages often display large, multi‐branched shapes rich in pseudopods, a structural adaptation that facilitates the engulfment of inhaled particles.^[^ ^32^ ^]^ This morphology differs from that of skin macrophages, which specialize in capturing surface antigens, or the shorter and spindle‐shaped forms typical of intestinal and cardiac macrophages.^[^ ^33^, ^34^ ^]^ Collectively, these features illustrate how the lung microenvironment has evolutionarily shaped pulmonary macrophages into highly specialized sentinels optimized for preserving gas exchange while mounting rapid responses to pathological damages.

Lung infection progresses through different stages—the acute phase, the pathogen clearance phase, and the inflammation resolution phase. In the acute phase, macrophages adopt a proinflammatory state that acts as an “accelerator” to control pathogens and initiate inflammation.^[^ ^27^ ^]^ As infection progresses into the pathogen clearance phase and the subsequent inflammation resolution phase, macrophages shift toward an anti‐inflammatory, tissue‐repairing state that functions as a “brake,” promoting tissue healing, maintaining barrier integrity, and restoring homeostasis.^[^ ^14^, ^35^ ^]^ The transition of macrophages from proinflammatory to anti‐inflammatory states is driven by signals in the pulmonary microenvironment, such as anti‐inflammatory cytokines such as IL‐10 and TGF‐β.^[^ ^16^, ^36^ ^]^ Additionally, the recruitment of monocytes and their differentiation into macrophages are involved in regulating inflammation and homeostasis in lung infections. However, in chronic pulmonary infections, the failure to resolve inflammation leads to a persistent immune response. Macrophages remain in a sustained proinflammatory state and continuously produce cytokines and ROS, which can result in tissue damage and fibrosis.^[^ ^37^ ^]^ Disturbance of the equilibrium between proinflammatory and anti‐inflammatory macrophages is a key driver of chronic lung disease pathogenesis.^[^ ^27^ ^]^ Understanding the dynamic shifts in macrophage functions throughout infection stages is crucial for developing targeted therapies to improve lung health.

### 2.2 Distinct Biology of Alveolar and Interstitial Macrophages at Steady State and During Infection

AMs are characterized by distinct phenotypic signatures. In mice, AMs typically express MerTK, CD64, CD68, CD206, and F4/80, while in the steady state, AMs lack CX_3_CR1 and CD11b but display high levels of CD11c and Siglec‐F. In humans, AMs are defined by the expression of CD206, CD169, CD11c, CD163, and MARCO, a profile largely conserved in non‐human primates. AMs exhibit heterogeneous spatial distributions within the lung, with studies showing that approximately 60% of alveoli lack resident AMs.^[^ ^2^, ^38^ ^]^ Only a minority (∼10%) remains stably anchored to the alveolar epithelium, where they form connexin 43 (Cx43)‐mediated gap junctions that enable direct intercellular communication. Notably, this sessile population persists even during inflammatory challenges such as lipopolysaccharide (LPS) exposure, coordinating immunoregulatory signals through synchronized calcium (Ca^2+^) waves propagated via the epithelial network. This mechanism is thought to dampen excessive inflammation and maintain tissue homeostasis.^[^ ^38^ ^]^ In contrast, the majority of AMs are highly motile and actively patrol the alveolar surface under steady state conditions. These cells migrate across adjacent alveoli by traversing the pores of Kohn, which allows for short‐range locomotion. Through this dynamic surveillance, migratory AMs contribute to the clearance of inhaled particles and cellular debris, thereby supporting immune defense.^[^ ^2^ ^]^

Interstitial macrophages (IMs) are a distinct lung‐resident population marked by SiglecF^−^CD11b^+^. They originate predominantly from postnatal Ly6C^+^ monocytes, with their recruitment and differentiation are largely driven by pathogens or inflammatory stimuli.^[^ ^39^ ^]^ Single‐cell technologies have resolved IMs into two to three distinct subpopulations.^[^ ^10^, ^12^ ^]^ Gibbings et al. identified three IM subsets based on CD11c and MHC class II expression: CD11c^lo^MHC II^lo^, CD11c^lo^MHC II^hi^, and CD11c^hi^MHC II^hi^.^[^ ^10^ ^]^ In contrast, Chakarov et al. described two conserved subsets across organs and species: Lyve1^lo^MHC II^hi^CX_3_CR1^hi^ and Lyve1^hi^MHC II^lo^CX_3_CR1^lo^. Spatial localization analysis via immunofluorescence further revealed that Lyve1^lo^MHC II^hi^ IMs are enriched in perineural regions, whereas Lyve1^hi^MHC II^lo^ IMs are predominantly situated near blood vessels. These subsets exhibit distinct functions. Lyve1^hi^MHC II^lo^ IMs restrain inflammation and fibrosis, as their depletion in pulmonary fibrosis models leads to increased vascular leakage, immune infiltration, and collagen deposition. In contrast, Lyve1^lo^MHC II^hi^ IMs display strong antigen‐presenting capacity.^[^ ^12^ ^]^

Macrophages act not only as sensors that perceive and respond to microenvironmental changes by adopting distinct phenotypes, but also as effectors that orchestrate critical immune functions. Their dynamic transitions across infection phases, from homeostasis to inflammation and resolution, underscore their indispensable roles in pulmonary immune regulation.

#### 2.2.1 The Acute Phase of Infection

During the acute phase of infection, the alveolar microenvironment undergoes rapid and profound changes, which are often referred to as the “pathogen‐induced damage phase.” ^[^ ^40^, ^41^ ^]^ It is characterized by a combination of intense immune activation, inflammatory responses, and substantial metabolic reprogramming within host cells.^[^ ^4^, ^42^ ^]^ Pathogens employ diverse strategies to manipulate the host immune system, including the secretion of small RNAs (sRNAs), to dampen the host's early immune response via interference with key pathways, such as blocking RIG‐I and IRAK1‐mediated type I interferon production.^[^ ^43^, ^44^, ^45^ ^]^ By targeting key sensors and regulators of the host immune system, pathogenic sRNAs disrupt the host immune response, thereby promoting pathogen survival.^[^ ^44^, ^45^ ^]^

Concurrently, the acute phase induces profound metabolic reprogramming in macrophages, resembling the Warburg effect observed in cancer cells. This metabolic shift is characterized by increased glycolysis, even in the presence of sufficient oxygen, and alterations in mitochondrial function.^[^ ^41^, ^46^, ^47^ ^]^ Within macrophages, this metabolic shift exhibits a functional dichotomy: intracellular pathogens co‐opt glycolytic intermediates for the biosynthesis of lipids, amino acids, and nucleotides, promoting their replication, whereas macrophages paradoxically leverage this metabolic state to enhance anti‐pathogen defenses.^[^ ^41^, ^48^, ^49^ ^]^ The Ras‐ERK‐PI3K‐mTOR signaling axis orchestrates these metabolic shifts by stabilizing HIF‐1α, a key transcription factor that drives glycolytic enzyme expression through mTORC1 signaling.^[^ ^50^, ^51^, ^52^, ^53^ ^]^ This metabolic reprogramming directs pyruvate toward lactate and acetyl‐CoA production, fostering an energetic environment that supports phagosomal acidification and pathogen containment.^[^ ^40^, ^54^ ^]^

#### 2.2.2 The Pathogen Clearance Phase

As the immune response transitions from the acute phase to the pathogen clearance phase, the focus shifts to the coordinated resolution of infection. This phase marked by the activation and dynamic interactions of various immune cell populations working in concert to eliminate pathogens.^[^ ^55^ ^]^ Macrophages not only serve as phagocytic effectors in pathogen clearance but also regulate the functions of other immune cells, including neutrophils, T cells, dendritic cells (DCs), and B cells.^[^ ^23^, ^55^, ^56^, ^57^, ^58^ ^]^ The precise orchestration of these cellular responses, along with the metabolic regulation governing immune cell activation, is essential for effective pathogen clearance.

AMs, which reside in the airway lumen, continue to phagocytose residual pathogens and debris,^[^ ^59^ ^]^ and secrete TGF‐β to induce CD4⁺ T cell anergy and foster FoxP3⁺ regulatory T cell differentiation, thereby dampening excessive inflammation.^[^ ^60^ ^]^ In the lung interstitium, interstitial macrophages (IMs) complement these functions by presenting antigens via high MHC II expression and fine‐tuning T helper responses.^[^ ^61^ ^]^ IMs can promote Th2 polarization in murine models and deliver costimulatory signals to tissue‐resident memory CD4⁺ T cells in humans, yet also restrain hyperactive Th2/Th17 responses through IL‐10 secretion.^[^ ^61^, ^62^ ^]^ Interestingly, compared with CD11b^+^ conventional dendritic cells (cDCs) and AMs, IMs induce a higher percentage of CD4^+^ T cells expressing FoxP3, a marker of regulatory T cells, particularly in models of allergic airway inflammation.^[^ ^63^ ^]^ This IL‐10‐ and TGF‐β‐dependent process highlights the potential of IMs in regulating immune responses during inflammation.^[^ ^63^, ^64^ ^]^ CD4⁺ T cells frequently colocalize with CX_3_CR1^+^/MHC II^+^ IMs, suggesting that IMs spatially regulate adaptive responses under inflammatory conditions.^[^ ^64^ ^]^

Concomitantly, circulating monocytes are recruited to the infected tissue, where inositol 1,4,5‐trisphosphate (IP_3_) and elevated intracellular calcium (Ca^2+^) fluxes drive their activation, migration, and differentiation into macrophages.^[^ ^65^, ^66^ ^]^ While these Ca^2^⁺ signals are indispensable for pathogen uptake and clearance, their dysregulation can perpetuate inflammation, compromise epithelial barriers, and exacerbate lung damage.^[^ ^58^ ^]^ Furthermore, macrophage population expansion is also driven by local proliferation. While the cell cycle of infected macrophages is arrested, uninfected bystander macrophages are triggered to proliferate, leading to macrophage accumulation.^[^ ^67^ ^]^

In viral infections like severe acute respiratory syndrome coronavirus 2 (SARS‐CoV‐2), macrophages orchestrate adaptive immunity through reciprocal crosstalk with *CD160*⁺*CD8*⁺ T cells. They interact with *CD160*⁺*CD8*⁺ T cells, which express chemokine receptors CXCR3 and CXCR6 that direct their migration to infected sites.^[^ ^35^, ^68^ ^]^ These T cells, which are rich in granzymes, cooperate with macrophages to eliminate infected cells.^[^ ^69^ ^]^ Moreover, *CD160*⁺*CD8*⁺ T cells secrete CCL5, which recruits additional macrophages, thus amplifying the antiviral response. At the same time, macrophages help regulate neutrophil activity by clearing apoptotic neutrophils and modulating the inflammatory environment, ensuring a balanced immune response. Notably, *Slamf9* ^+^ macrophages co‐express ligand‐receptor pairs such as *Pecam1*/*Cd38*, *Ccl5*/*Ccr5*, and *Ccl8*/*Ccr1*, as detected by spatial enhanced resolution omics‐sequencing (Stereo‐seq), suggesting that they actively participate in neutrophil recruitment and modulation of their function.^[^ ^14^, ^70^ ^]^ Similarly, macrophage interactions with DCs, which are critical for initiating adaptive immunity, further contribute to the efficient coordination of immune responses.^[^ ^35^ ^]^

Furthermore, macrophages play crucial roles in regulating immune cell function in specific regions of the lung, such as the adventitia of pulmonary vessels, where regional immune hubs are located.^[^ ^71^ ^]^ FRβ^+^ IMs predominate in these areas and are key in maintaining local immune homeostasis. These macrophages express chemokines such as CXCL13 and CCL8, which recruit B cells, Th2 cells, group 2 innate lymphoid cells (ILC2s), and CD103^+^ DCs.^[^ ^72^, ^73^ ^]^ Through these interactions, FRβ^+^ IMs help coordinate immune responses, ensuring that immune cells are appropriately positioned to clear the infection while minimizing inflammatory damage.^[^ ^17^ ^]^

#### 2.2.3 The Inflammation Resolution Phase

As the immune response progresses to the inflammation resolution phase, the lung microenvironment undergoes a critical transition from a proinflammatory state to an anti‐inflammatory state, which is essential for restoring tissue homeostasis and facilitating repair.^[^ ^74^ ^]^ As inflammation subsides, immune cell activation decreases, proinflammatory cytokines are reduced, and tissue repair mechanisms are initiated.^[^ ^75^, ^76^ ^]^ Macrophages contribute significantly to this resolution by regulating inflammatory mediators and supporting tissue regeneration.

The resolution of inflammation relies on a carefully coordinated process where macrophages reduce ROS and proinflammatory cytokines while simultaneously promoting the accumulation of anti‐inflammatory signals.^[^ ^77^ ^]^ Moreover, macrophages play a critical role in clearing apoptotic cells, such as neutrophils and T cells, thus preventing excessive inflammation and facilitating efficient tissue repair.^[^ ^78^, ^79^ ^]^ This process, known as efferocytosis, not only prevents the release of proinflammatory molecules and toxins but also triggers the secretion of reparative mediators such as TGF‐β, PAF, and PGE2. These mediators suppress proinflammatory cytokines (e.g., TNF‐α) and chemokines (e.g., MIP‐2), further promoting tissue repair.^[^ ^75^, ^80^, ^81^, ^82^ ^]^

As resolution progresses, macrophages shift phenotypes to support tissue repair. For example, after SARS‐CoV‐2 infection, the number of *S100a9* ^+^ and *Slamf9* ^+^ macrophages in alveolar DC–T immunity hubs decreases. These macrophages differentiate into *Trem2* ^+^ immune regulatory macrophages and *Fbp1* ^+^ proliferating macrophages, which contribute to repair by secreting anti‐inflammatory cytokines and clearing residual pathogens and dead cells.^[^ ^14^, ^35^ ^]^ In addition, macrophage interactions with regulatory T cells (Tregs), which dampen inflammation, further facilitate resolution. Tregs exert pro‐resolution effects both directly and indirectly through macrophages, decreasing proinflammatory cytokine secretion and increasing the levels of reparative factors like TGF‐β.^[^ ^76^, ^83^, ^84^ ^]^

Another key feature of lung repair is the transient appearance of cytokeratin 8 (KRT8)^hi^ transitional epithelial progenitor cells. However, their prolonged persistence or dysregulation can disrupt alveolar architecture and drive fibrotic remodeling, contributing to long‐term complications such as post‐acute sequelae of COVID‐19 (PASC).^[^ ^85^, ^86^ ^]^ Emerging evidence has revealed a critical role for macrophage peroxisomes in orchestrating alveolar recovery and mitigating fibrotic outcomes. By modulating lipid metabolism, peroxisomes preserve mitochondrial function and support macrophage programs that promote alveolar type 2 (AT2) cells renewal.^[^ ^37^ ^]^ However, sustained interferon signaling, particularly IFN‐γ signaling, can impair this process by suppressing peroxisome biogenesis and triggering pexophagy (autophagy of peroxisomes), ultimately compromising the reparative functions of macrophages.^[^ ^87^ ^]^ Pharmacological enhancement of peroxisome activity, such as through sodium 4‐phenylbutyrate (4‐PBA), has shown promise in restoring macrophage‐mediated repair and limiting long‐term lung damage.^[^ ^37^ ^]^

The resolution of inflammation is typically an orderly and protective process; however, defects in this pathway can lead to chronic inflammation (e.g., COPD, asthma, and pulmonary fibrosis).^[^ ^37^, ^88^, ^89^, ^90^ ^]^ Impaired phagocytosis, excessive apoptosis, or reduced specialized pro‐resolving mediators (SPMs) production can all hinder proper resolution, resulting in persistent inflammation and tissue damage.^[^ ^91^ ^]^ A deeper understanding of how targeting macrophages to modulate these molecular pathways could offer potential therapeutic strategies for improving outcomes in pulmonary infections and preventing long‐term lung damage.

### 2.3 Metabolic Cues Shape Macrophage Plasticity in Pulmonary Infections

Metabolic regulation has emerged as a critical determinant of macrophage function in the lung, particularly during pulmonary infections. Both systemic and local metabolic cues, ranging from host‐derived to microbiota‐generated metabolites, profoundly shape macrophage phenotype, gene expression, and immune responsiveness, thereby influencing the outcome of respiratory diseases.^[^ ^92^, ^93^ ^]^

Obesity exemplifies how systemic metabolic dysregulation alters pulmonary immunity. Elevated levels of the adipokine leptin in obese individuals impair antiviral immunity by reprogramming macrophages, particularly in bronchoalveolar lavage (BAL) macrophages.^[^ ^93^, ^94^ ^]^ AMs, which uniquely express the leptin receptor (*Lepr*), are particularly sensitive to such metabolic cues. Leptin signaling exerts dual effects on these cells.^[^ ^93^ ^]^ On the one hand, it undermines antiviral defenses by suppressing protein kinase R (PKR) expression and the production of interferon‐β (IFN‐β) or interferon‐stimulated genes (ISGs), such as 2′‐5′‐oligoadenylate synthetase (*OAS*).^[^ ^93^ ^]^ This inhibition is reinforced by the induction of suppressor of cytokine signaling 3 (*SOCS3*), which blocks JAK/STAT signaling to weaken host resistance to viral infection.^[^ ^94^, ^95^ ^]^ On the other hand, LEP‐R signaling protects AMs from necroptosis to reduce the release of interleukin‐1α that prevents neutrophil recruitment and consequent tissue injury.^[^ ^93^ ^]^ Thus, hyperleptinemia creates a complex immunometabolic state in which macrophage survival and tissue integrity are preserved at the cost of compromised antiviral capacity.

In addition to host‐derived metabolites, the gut microbiota and its metabolic by‐products—particularly short‐chain fatty acids (SCFAs)—also significantly influence macrophage function in the lung.^[^ ^19^, ^96^ ^]^ SCFAs, including acetate, propionate, and butyrate, have been linked to shifts in macrophage function, characterized by a reduction in mTOR kinase activity, increased microtubule‐associated protein 1 light chain 3 alpha (LC3)‐associated host defense, and increased production of antimicrobial peptides, without provoking excessive proinflammatory cytokine production.^[^ ^97^, ^98^, ^99^, ^100^ ^]^ Mechanistically, butyrate has been shown to inhibit histone deacetylase 3 (HDAC3) activity, leading to acetylation and suppression of STAT1 phosphorylation and dimerization, thereby steering macrophages toward an anti‐inflammatory state that confers protection against Methicillin‐resistance *Staphylococcus aureus* (MRSA)‐induced pneumonia.^[^ ^101^ ^]^ These effects are not limited to local immune modulation; SCFAs also affect hematopoietic precursors in the bone marrow, promoting the generation of macrophage and dendritic cell progenitors, thus altering the composition of immune cells recruited to the lung.^[^ ^92^ ^]^

Dietary habits further influence this process by shaping the gut microbial landscape (e.g., the ratio of *Firmicutes* to *Bacteroidetes*) and SCFA output.^[^ ^102^, ^103^ ^]^ High‐fiber diets increase SCFA production and have been associated with protection against allergic airway inflammation, whereas low‐fiber diets lead to reduced SCFA levels and heightened disease susceptibility.^[^ ^104^ ^]^ Importantly, SCFAs regulate key metabolic pathways within macrophages, including the balance between glycolysis and oxidative phosphorylation, and the promotion of fatty acid oxidation.^[^ ^105^ ^]^ These metabolic changes not only fuel macrophage activity but also influence transcriptional programs that guide their differentiation and functional polarization.

Together, these findings underscore the profound role of metabolic signals, both host‐ and microbiota‐derived, in orchestrating macrophage plasticity in the lung. By reprogramming cellular metabolism, these cues determine macrophage fate, functional phenotype, and their ability to either resolve or exacerbate infection‐driven inflammation.

## 3 Single‐Cell Profiling of Macrophage Subtypes in Pulmonary Infections

Single‐cell transcriptomics has uncovered a remarkable diversity of macrophage states across infectious and inflammatory diseases. A central challenge is how to organize these transcriptional states into biologically meaningful categories that are reproducible and functionally coherent. In infectious contexts, numerous single‐cell studies have delineated macrophage heterogeneity across pathogens. For example, analysis of murine lungs infected with *Mtb*,^[^ ^106^ ^]^ or bronchoalveolar lavage macrophages from patients with latent versus active tuberculosis,^[^ ^107^ ^]^ or *ex vivo* infection of human lung tissue with SARS‐CoV‐2,^[^ ^57^, ^108^, ^109^ ^]^ and spatiotemporal mapping of *Slamf9* ^+^ macrophages during coronavirus infection ^[^ ^14^, ^35^ ^]^ have shown distinct macrophage subclusters. Comparable findings have also emerged in influenza, *Streptococcus pneumoniae*, and other models, underscoring the generalizability of these observations. Across studies, the subsets are characterized by recurring transcriptional programs such as interferon and TNF‐driven inflammatory modules, chemokine expression patterns, metabolic remodeling signatures, cell‐cycle–linked proliferative states, and markers of senescence. They are further distinguished by spatial localization to niches such as immunity hubs or interstitial compartments, and by dynamic transitions across the course of infection, from acute inflammation to resolution.

Parallel efforts in cancer immunology have similar insights. According to single‐cell and spatial multi‐omics data across multiple tumor types, a conserved taxonomy of tumor‐associated macrophages (TAMs) was proposed. This framework delineates seven major classes, including interferon‐primed, inflammatory cytokine‐enriched, immune‐regulatory, lipid‐associated, pro‐angiogenic, resident tissue–like, and proliferating TAMs. Each category is anchored by a characteristic transcriptional program, such as ISG/IDO modules in interferon‐primed TAMs, IL1B/CCL chemokine clusters in inflammatory TAMs, ARG1/MRC1 and efferocytosis signatures in regulatory TAMs, APOE/APOC modules in lipid‐associated TAMs, VEGFA/SPP1 programs in angiogenic TAMs, scavenger and tissue‐specific markers in resident‐like TAMs, and MKI67‐driven cell‐cycle genes in proliferating TAMs. Importantly, this framework emphasized recurrence and functional coherence, helping to link macrophage programs to therapeutic targets such as TREM2, MARCO, and QPCTL.^[^ ^110^ ^]^

**Figure 2:**
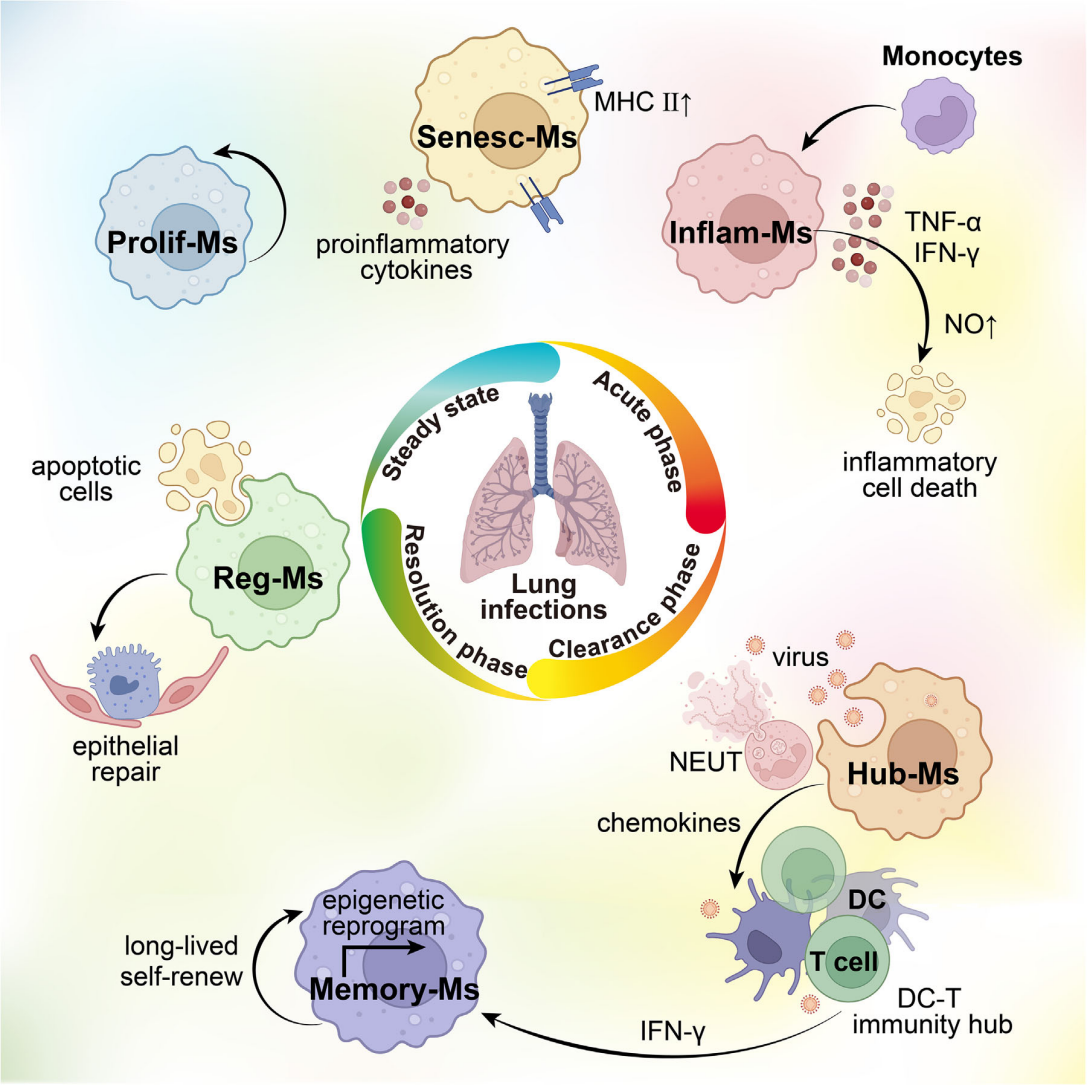
Macrophage subtypes during different stages of pulmonary infection and their functional roles. Inflam‐Ms, inflammatory cytokine‐enriched macrophages; Hub‐Ms, immunity hubs‐associated macrophages; Reg‐Ms, immune regulatory macrophages; Prolif‐Ms, proliferating macrophages; Memory‐Ms, Memory macrophages; Senesc‐Ms, Senescent macrophages; DC, dendritic cell; NEUT, neutrophil. Created with BioRender.com.

For pulmonary infections, six macrophage subtypes are proposed: inflammatory cytokine‐enriched macrophages (Inflam‐Ms), immunity hubs–associated macrophages (Hub‐Ms), immune regulatory macrophages (Reg‐Ms), proliferating macrophages (Prolif‐Ms), memory macrophages (Memory‐Ms), and senescent macrophages (Senesc‐Ms) (Figures 1 and **2**; Table 1). This framework rests on three principles: i) recurrence across independent scRNA‐seq datasets of viral, bacterial, and fungal infections; ii) functional coherence such as cytokine production, spatial coordination, efferocytosis, proliferation, epigenetic reprogramming, and senescence; and iii) orthogonal validation through spatial mapping, cell‐cycle or senescence markers, and epigenetic profiling, *etc*. By integrating single‐cell signatures with function, this six‐subtype model provides a reproducible and mechanistically interpretable scaffold for understanding macrophage plasticity in the infected lung.

### 3.1 Inflammatory Cytokine‐Enriched Macrophages

Inflammatory cytokine‐enriched macrophages (Inflam‐Ms) play pivotal roles during pulmonary infections. Inflam‐Ms are activated by type I interferons and proinflammatory cytokines, which reprogram their functional state and metabolism. The release of inflammatory cytokines by Inflam‐Ms is critical for effective pathogen clearance; however, excessive cytokines can lead to tissue damage, while an insufficient inflammatory response allows pathogens to evade immune surveillance.^[^ ^55^, ^111^ ^]^ Notably, Inflam‐Ms produce distinct responses when confronted with various types of infections, and their gene expression profiles are characteristic of the specific pathogens.

During the progression of SARS‐CoV‐2 infection, *DDX60* ^+^ *CHIT1* ^hi^ Inflam‐Ms are significantly elevated during the acute phase. In this stage, Inflam‐Ms increases expression of IFN‐responsive genes such as *OAS1*, *ISG15*, and *RSAD2*. As infection progresses, *DDX60* ^+^ *CHIT1* ^hi^ Inflam‐Ms increase expression of key inflammatory markers, including *HLA‐DRB1*, *MRC1*, and *SERPINE2*.^[^ ^15^ ^]^ In severe cases of coronavirus disease 2019 (COVID‐19), excessive production of proinflammatory cytokines and complement components drives a life‐threatening cytokine storm, leading to diffuse alveolar damage and acute respiratory distress syndrome (ARDS).^[^ ^55^, ^111^ ^]^ As an intrinsic feature of ARDS, hypoxia further drives the release of soluble complement C5a.^[^ ^112^ ^]^ Elevated C5a levels correlate with disease severity and, via the C5a–C5aR1 axis, induce CCL2 secretion to recruit monocytes and promote their differentiation into C5a receptor 1^+^ (C5aR1^+^) Inflam‐Ms.^[^ ^113^, ^114^ ^]^ This feed‐forward loop sustains and exacerbates lung inflammation. In Inflam‐Ms, an overactive immune response is driven by elevated levels of cytokines such as TNF‐α and IFN‐γ, which also induce PANoptosis, a unique form of inflammatory cell death.^[^ ^115^ ^]^

In certain chronic granulomatous infections, such as those caused by *Mycobacterium tuberculosis*, Inflam‐Ms exhibit distinct response patterns. They prefer to utilize oxidative stress and express a specific inflammatory molecular profile that does not primarily focus on interferons to combat the infection. In the context of *Mtb* infection, Inflam‐Ms are primarily derived from monocytes and exhibit a phagocytic phenotype, which is characterized primarily by *Nos2* expression.^[^ ^106^ ^]^ *Nos2* ^+^ Inflam‐Ms also express high levels of proinflammatory genes, including *Clec4e* (Mincle) and *Saa3* (serum amyloid A),^[^ ^106^, ^116^ ^]^ which increase the production of ROS and reactive nitrogen species (RNS) to promote macrophage clearance of *Mtb*.^[^ ^117^ ^]^ *Nos2* ^+^ Inflam‐Ms upregulate glycolysis and pathways involved in RNS production, and this metabolic shift ensures persistent proinflammation during infection.^[^ ^118^ ^]^ During active tuberculosis, monocyte‐derived *FCN1* ^+^ Inflam‐Ms become enriched in signaling pathways associated with TLR signaling, the lipopolysaccharide (LPS) response, TNF signaling, the IL‐1 response, and interferon pathways.^[^ ^107^ ^]^ Upon activation by pathogen‐associated molecular patterns (PAMPs) via TLR4 and TLR2, these macrophages promote ROS production in a Nox2‐dependent manner.^[^ ^119^ ^]^ Compared to SARS‐CoV‐2, Inflam‐Ms infected with *Mtb* display a lower and more sustained level of inflammatory response.^[^ ^120^ ^]^ Moreover, to evade host immune defenses, *Mtb* can produce the phosphatase PtpB that directly targets and alters the composition of host membrane phospholipids, thereby inhibiting pyroptosis. By hijacking host ubiquitin, PtpB prevents the activation of gasdermin D (GSDMD), a key mediator of pyroptotic cell death.^[^ ^121^, ^122^, ^123^ ^]^ Disruption of PtpB activity could enhance GSDMD‐dependent immune responses, leading to reduced intracellular pathogen survival.^[^ ^123^ ^]^

### 3.2 Immunity Hubs‐Associated Macrophages

**Figure 3:**
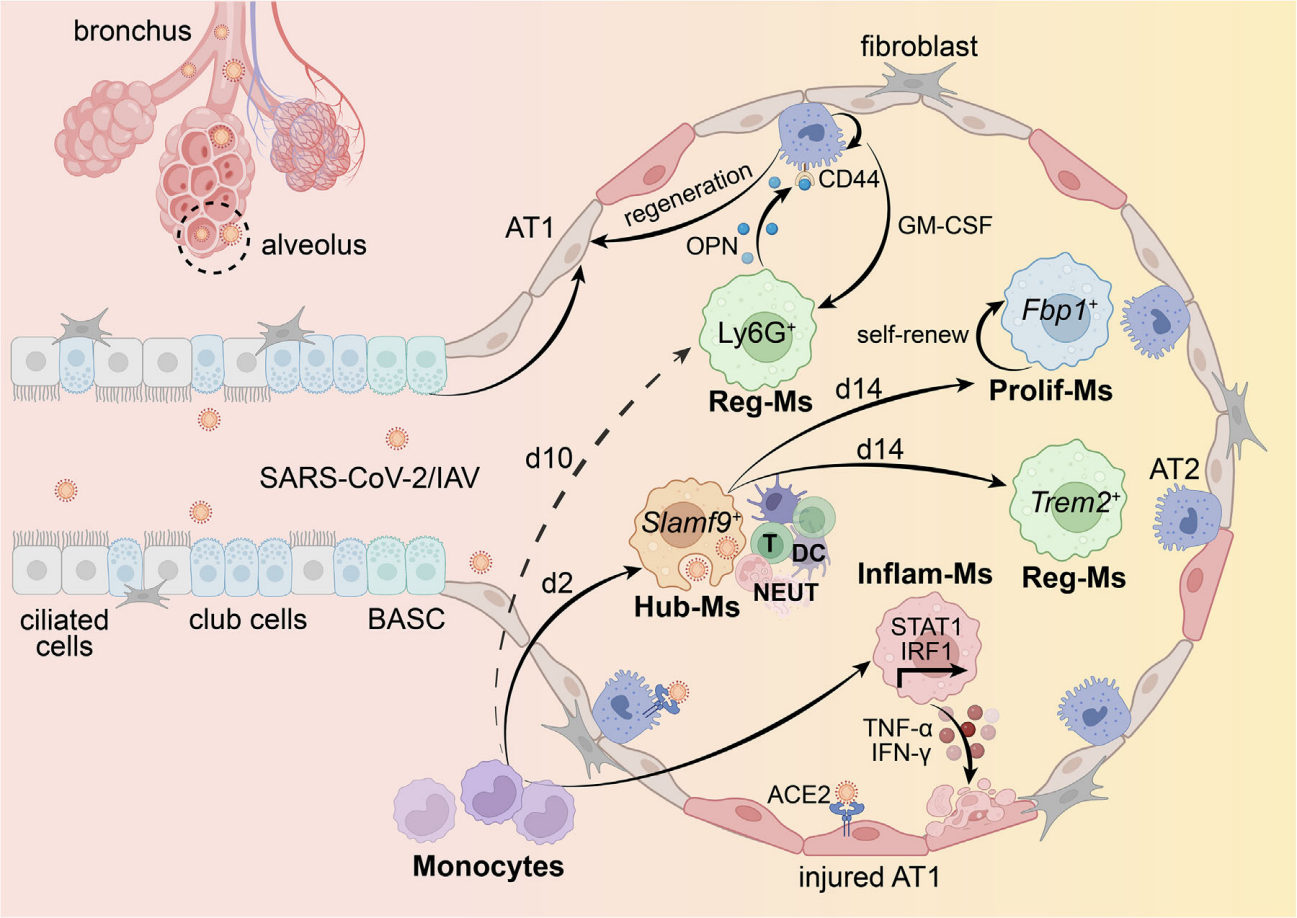
Functions and transitions of macrophage subtypes within the alveolar microenvironment during SARS‐CoV‐2 or influenza A virus (IAV) infection. Inflam‐Ms, inflammatory cytokine‐enriched macrophages; Hub‐Ms, immunity hubs‐associated macrophages; Reg‐Ms, immune regulatory macrophages; Prolif‐Ms, proliferating macrophages; AT1, alveolar type 1 cells; AT2, alveolar type 2 cells; BASCs, bronchoalveolar stem cells; OPN, osteopontin; DC, dendritic cell; NEUT, neutrophil. Created with BioRender.com.

Immunity hubs‐associated macrophages (Hub‐Ms) can be defined by their consistent spatial enrichment within alveolar dendritic cell–T cell immunity hubs and by a characteristic transcriptional and receptor–ligand program that enables intensive crosstalk with DCs, T cells, and neutrophils. Direct evidence comes from Stereo‐seq–based spatiotemporal mapping of infected lungs, showing a preferential accumulation of a *Slamf9* ^+^ monocyte‐derived subset. *Slamf9* ^+^ Hub‐Ms, distinguished by heightened viral detection rates and sustained proliferative activity, are localized within hubs enriched for *Ccr7* ^+^ *Ido1* ^+^ DCs, *Cd160* ^+^CD8^+^ T cells, and *Tnfrsf4* ^+^CD4^+^ T cells.^[^ ^14^, ^35^, ^124^, ^125^ ^]^ Functionally, they phagocytose viral particles and interact with *Isg12* ^+^ *Cst7* ^+^ neutrophils through receptor–ligand axes such as PECAM1, CCL5/8, CD80, and IL‐10, while secreting chemokines including CCL7, CCL8, and CCL13 to recruit T cells.^[^ ^14^, ^126^, ^127^ ^]^ During SARS‐CoV‐2 infection, *Slamf9* ^+^ Hub‐Ms expand rapidly within these hubs, orchestrating immune cell cooperation and making a decisive contribution to viral clearance. Once infection is resolved, their population contracts to baseline levels, but by day 14 post‐infection, they undergo functional diversification, giving rise to *Trem2* ^+^ Reg‐Ms and *Fbp1* ^+^ Prolif‐Ms that promote inflammation resolution and tissue repair ^[^ ^14^ ^]^ (**Figure** **3**).

Insights from the tumor microenvironment further underscore the broader immunological significance of hub structures. Recent spatial transcriptomic analyses in human lung cancer have identified “stem‐immunity hubs,” enriched for *TCF7* ^+^ *PD‐1* ^+^ stem‐like CD8^+^ T cells, *CCR7* ^+^ *LAMP3* ^+^ DCs, and *CCL19* ^+^ fibroblasts, which correlate with favorable responses to immunotherapy. Within these hubs, *CXCL10* ^+^ macrophages preferentially colocalize with effector CD8⁺ T cells and reinforce cytotoxic programs.^[^ ^131^ ^]^ These results emphasize that hub‐localized macrophages might function as active organizers of multicellular immunity or coordinate immune programs.

Beyond the *Slamf9* ^+^ subset, multiple single‐cell studies have described macrophage states with features strongly suggestive of hub association, even though their localization has not yet been directly demonstrated. For example, *IL‐18* ^hi^ subsets capable of antigen cross‐presentation drive the expansion of CD103^+^CD8^+^ resident memory T cells (T_RM_) after influenza infection.^[^ ^128^, ^129^ ^]^ Similarly, monocyte‐derived *CCL23* ^hi^ macrophages identified in *Mtb* infection express chemokines that preferentially recruit CD8^+^CCR6^+^ T cells via the CCL20–CCR6 axis, implying a role in organizing localized immunity hubs.^[^ ^107^ ^]^ Single‐cell profiling of *Pseudomonas aeruginosa* infection likewise revealed infection‐stage–dependent macrophage chemokine modules, including CXCL4/PF4 and CXCL16, which coordinate NK, NKT, and T‐cell responses in a manner consistent with hub biology during chronic bacterial inflammation.^[^ ^130^ ^]^ However, Hub‐Ms beyond the *Slamf9* ^+^ subset remain poorly characterized, and their spatial organization, ontogeny, and conserved molecular signatures require further elucidation. Future studies integrating spatial transcriptomics, lineage tracing, and interactome analyses will be crucial to define their identity and functions. Such knowledge could inform new strategies to enhance hub‐mediated immune coordination during infections.

### 3.3 Immune Regulatory Macrophages

Immune regulatory macrophages (Reg‐Ms) act as crucial modulators in the host immune response to infection. On one hand, their ability to suppress excessive inflammation plays a protective role by preventing cytokine storms and minimizing tissue damage.^[^ ^80^, ^132^ ^]^ On the other hand, insufficient or dysregulated activity of Reg‐Ms may compromise pathogen clearance, potentially resulting in unresolved inflammation and chronic infection. In addition to their immunomodulatory functions, Reg‐Ms are actively involved in tissue repair following infection. They facilitate epithelial regeneration, extracellular matrix remodeling, and angiogenesis, thereby contributing to the restoration of tissue integrity. These reparative processes are underpinned by distinct phenotypic changes, such as cytoskeletal reorganization, enhanced motility, and metabolic reprogramming, all of which are essential for efficient tissue regeneration.^[^ ^133^, ^134^ ^]^

A fundamental mechanism by which Reg‐Ms exert their immunoregulatory function is the clearance of apoptotic cells (efferocytosis), a process that not only removes proinflammatory cellular remnants but also delivers anti‐inflammatory signals. Macrophages initiate efferocytosis by sensing exposed phosphatidylserine (PtdSer) on apoptotic cells through the receptor tyrosine kinases (RTKs) AXL and MERTK, which require the bridging proteins growth arrest–specific 6 (GAS6) or protein S alpha (PROS1) for ligand binding.^[^ ^135^ ^]^ Critically, full activation of the anti‐inflammatory tissue‐repair phenotype requires a second signal: the concurrent presence of IL‐4 or IL‐13 together with apoptotic targets drives the characteristic transcriptional and metabolic reprogramming of Reg‐Ms.^[^ ^136^ ^]^ Under these dual stimuli, macrophages up‐regulate key repair genes (e.g., *Arg1*, *Retnla*, *Chil3*) and redirect L‐arginine metabolism toward ornithine and polyamines, fostering collagen synthesis and extracellular matrix remodeling.^[^ ^137^, ^138^ ^]^ Simultaneously, ARG1 induction supports a shift to oxidative metabolism, marked by elevated fatty‐acid oxidation and mitochondrial respiration, which suppresses proinflammatory cytokine release and promotes the secretion of pro‐resolving mediators.^[^ ^139^, ^140^ ^]^

Reg‐Ms also promote tissue repair during the resolution phase. During infection with influenza A virus (IAV), circulating monocytes are recruited into the alveolar niche via CCR2‐dependent signaling pathways.^[^ ^141^, ^142^ ^]^ Within this microenvironment, they are exposed to granulocyte–macrophage colony‐stimulating factor (GM‐CSF) secreted by adjacent alveolar type 2 (AT2) epithelial cells.^[^ ^143^ ^]^ *Ex vivo* studies have shown that upon GM‐CSF stimulation, these monocyte‐derived precursors markedly increase the expression of *lymphocyte antigen 6 complex, locus G* (*Ly6g*). In vivo, signaling through the GM‐CSF receptor is essential not only for their differentiation into Ly6G⁺ Reg‐Ms but also for the upregulation of arginase‐1 (ARG1) following IAV infection. Once differentiated, Ly6G⁺ Reg‐Ms undergo rapid expansion, peaking at approximately day 10 post‐infection and disappearing by day 17.^[^ ^141^ ^]^ Activation of the IL‐4 receptor further stimulates Ly6G⁺ Reg‐Ms to produce osteopontin (OPN), a ligand for CD44, along with proinflammatory cytokines such as IL‐1 and TNF‐α—all of which have been shown to support alveolar regeneration.^[^ ^144^, ^145^ ^]^ Osteopontin binding to CD44^hi^ AT2 progenitors promotes their differentiation into type I alveolar (AT1) epithelial cells, thereby preventing abnormal bronchiolization and supporting effective alveolar regeneration.^[^ ^146^, ^147^ ^]^ Supporting this, *ex vivo* co‐culture experiments demonstrate that Ly6G⁺ Reg‐Ms directly facilitate AT2 wound closure, emphasizing their essential role in orchestrating epithelial repair during recovery from IAV infection ^[^ ^141^ ^]^ (Figure 3).

In the case of SARS‐CoV‐2 infection, single‐cell RNA sequencing has revealed a distinct subpopulation of Reg‐Ms characterized by high expression of *SPP1* and intermediate expression of *CHIT1* (*SPP1* ^hi^ *CHIT1* ^int^ Reg‐Ms).^[^ ^15^ ^]^ Notably, these *SPP1* ^hi^ *CHIT1* ^int^ Reg‐Ms upregulate genes associated with cholesterol metabolism and tissue remodeling, such as peroxisome proliferator‐activated receptors (PPARs) and their downstream targets.^[^ ^148^, ^149^ ^]^ These *SPP1* ^hi^ *CHIT1* ^int^ Reg‐Ms are predominantly derived from infiltrating *DDX60* ^+^ *CHIT1* ^hi^ monocyte‐derived macrophages.^[^ ^15^, ^108^ ^]^ Moreover, a distinct subpopulation of *Trem2* ^+^ Reg‐Ms has been identified during the inflammation resolution phase in the lung following SARS‐CoV‐2 clearance. These *Trem2* ^+^ Reg‐Ms differentiate from monocyte‐derived *Slamf9* ^+^ Int‐Ms and are integral to the resolution of inflammation, immune homeostasis, and tissue repair.^[^ ^14^ ^]^

### 3.4 Proliferating Macrophages

In response to tissue injury or infection, proliferating macrophages (Prolif‐Ms) play a critical role in rapidly replenishing the macrophage pool, maintaining a dynamic balance between regulating the inflammatory response and promoting tissue repair. Prolif‐Ms also contribute to the renewal of tissue‐resident macrophages under steady‐state conditions.^[^ ^150^, ^151^ ^]^ The proliferation of macrophages is tightly regulated by specific signaling pathways.^[^ ^152^ ^]^ The surrounding microenvironment is also essential for controlling macrophage proliferation, with Prolif‐Ms relying on the collagen receptor LAIR1 (CD305) to sense stromal cues that influence their fitness and function.^[^ ^153^, ^154^ ^]^ This interaction is critical for both homeostasis and disease progression.

A critical aspect of macrophage proliferation is its regulation through specific signaling pathways. Among these, Wnt signaling promotes the stabilization of β‐catenin, which accumulates in the nucleus, where it interacts with transcription factors like TCF/LEF to activate downstream genes.^[^ ^155^ ^]^ In AMs, this pathway not only facilitates the production of inflammatory mediators but also plays a role in restricting excessive proliferation and maintaining stemness.^[^ ^156^ ^]^ Moreover, Wnt‐induced β‐catenin accumulation influences metabolic shifts, particularly through its interaction with hypoxia‐inducible factor 1‐alpha (HIF‐1α), a transcription factor that drives glycolysis while inhibiting mitochondrial respiration.^[^ ^157^, ^158^ ^]^ Interestingly, activation of Wnt signaling suppresses the stemness and self‐renewal of alveolar macrophages while simultaneously enhancing their proinflammatory function.^[^ ^152^ ^]^

The microenvironment in which macrophages reside is also a key determinant of their behavior, including their proliferative capacity. Macrophages, including both monocyte‐derived and tissue‐resident macrophages, are exposed to collagen‐rich niches, which provide both physical and biochemical cues that influence their function.^[^ ^159^ ^]^ Collagen receptors such as LAIR1, which are expressed on both monocytes and lung IMs, mediate interactions with collagen and other stromal proteins, which help regulate macrophage survival, proliferation, and differentiation.^[^ ^153^ ^]^

Pulmonary macrophages arise from yolk sac precursors and fetal liver monocytes, giving rise to two major lineages: alveolar macrophages (AMs) and interstitial macrophages (IMs).^[^ ^9^ ^]^ Over 90% of AMs maintain population stability primarily through self‐renewal, supported by alveolar niche factors such as GM‐CSF.^[^ ^9^ ^]^ By contrast, IMs, occupying the interstitial space, rely on a mixed strategy of local proliferation and continuous monocyte replenishment. In addition, IM subsets display different renewal patterns: CD206^+^ IMs near the alveolar wall predominantly self‐proliferate under GM‐CSF and TGF‐β signaling, whereas Ly6C^+^ IMs adjacent to vessels and bronchi are mainly replenished by CCL2‐ and M‐CSF–driven monocyte recruitment.^[^ ^160^, ^161^ ^]^ These differences highlight that AMs favor a self‐renewal to minimize barrier disruption, while IMs remain primed for rapid turnover in response to environmental cues.

Under conditions of acute inflammation or lung injury, these proliferative programs are dynamically reshaped. AMs are often depleted by pathogen uptake and efferocytosis, necessitating rapid replenishment from both monocyte influx and accelerated self‐proliferation. While monocyte‐derived AMs dominate during the acute phase, their self‐renewal program reestablishes long‐term stability once inflammation resolves. Similarly, IMs undergo activation and loss during infection and are replenished primarily through circulating monocytes differentiating into proinflammatory IMs, with CD206^+^ IMs contributing a smaller proliferative component.^[^ ^160^ ^]^ Together, orchestrated by both niche‐derived signals and inflammatory context, AMs and IMs employ distinct but complementary proliferative strategies to sustain lung immune homeostasis and repair.

Interestingly, during *Mtb* infection, macrophage proliferation is restricted to uninfected bystander cells, whereas infected macrophages undergo cell cycle arrest. Both lung‐resident AMs and IMs exhibit basal levels of self‐renewal even under uninfected conditions, as shown by Ki67 labeling.^[^ ^10^, ^162^ ^]^ Flow cytometry analyses show that *Ki67* and inducible nitric oxide synthase (*iNOS*) expression are mutually exclusive in AM and IM populations. *iNOS* expression is markedly upregulated in infected macrophages; however, these cells remain Ki67^−^, indicating that active proliferation does not occur in the infected cells themselves. In contrast, a subset of uninfected macrophages retains Ki67^+^.^[^ ^11^, ^163^ ^]^ These findings suggest that macrophage proliferation during *Mtb* infection is largely confined to uninfected bystander cells.

### 3.5 Memory Macrophages

Innate immunity was long regarded as incapable of memory. Recent advances have revealed that macrophages can acquire long‐lasting memory‐like states in response to infection or inflammatory cues.^[^ ^164^, ^165^ ^]^ This phenomenon, broadly referred to as trained immunity, results from persistent epigenetic and metabolic reprogramming, such as Warburg‐like metabolic shifts and chromatin remodeling, which together enhance pathogen recognition, cytokine secretion, and effector responses.^[^ ^3^, ^166^, ^167^ ^]^ Unlike adaptive immune memory, it is antigen‐independent, enabling macrophages to mount more effective responses upon subsequent encounters.^[^ ^3^, ^168^ ^]^ Memory macrophages (Memory‐Ms) primarily arise from the proliferation of resident macrophage pools and sustain immune competence independently of circulating monocytes.^[^ ^3^, ^169^ ^]^

scRNA‐seq and single‐nucleus ATAC sequencing (snATAC‐seq) analyses reveal that lung‐resident macrophages acquire distinct memory programs characterized by increased chromatin accessibility at interferon‐stimulated loci (e.g., *Ifnb1, Stat1, Ifit1, Mx1*) and activating histone modifications such as H3K4me1 and H3K27ac.^[^ ^3^, ^170^ ^]^ Developmentally, Memory‐Ms follow a two‐stage trajectory: their priming requires CD8^+^ T cell help through IFN‐γ and contact‐dependent interactions, while their persistence and recall responses can occur independently of T cell input.^[^ ^169^ ^]^ Notably, studies diverge on whether T cell help is essential for memory programming. For example, viral airway infection induces memory AMs through an early CD8^+^ T cell–dependent priming phase,^[^ ^169^ ^]^ whereas parenteral BCG vaccination generates memory AMs via the gut–lung axis independently of T cells or IFN‐γ.^[^ ^171^ ^]^ These discrepancies likely reflect differences in context: airway infection drives local, contact‐dependent priming by effector T cells, while BCG engages systemic immune–metabolic circuits that reprogram AMs through soluble mediators.

Functionally, Memory‐Ms broaden host defense beyond antigen‐specific mechanisms. Prior infections, including SARS‐CoV‐2, influenza A virus, *Staphylococcus aureus*, and *Mycobacterium tuberculosis*, induce durable epigenetic changes in airway‐resident macrophages.^[^ ^165^, ^167^, ^168^ ^]^ These include enhanced accessibility at interferon‐related effector genes (e.g., *Ifnb1*, *Stat1*, *Ifit1*) and ISG loci (e.g., *Mx1*, *Oas3*, *Cd86*, *Rsad2*), enabling faster antiviral responses upon reinfection.^[^ ^3^, ^170^ ^]^ Such “preconditioning” not only accelerates clearance of homologous pathogens but also confers heterologous protection.^[^ ^165^, ^167^ ^]^ For instance, prior SARS‐CoV‐2 infection has been shown to mitigate the severity of secondary influenza A virus infection,^[^ ^3^ ^]^ while chronic exposure to *Mtb* or other respiratory pathogens primes the lung to limit replication of subsequent invaders, including SARS‐CoV‐2.^[^ ^168^ ^]^ This cross‐protection is largely mediated by an enhanced antiviral state marked by elevated IFN‐I, TNF‐α, and IL‐1 production, which primes the lung for more effective immunity.^[^ ^3^, ^170^ ^]^ Importantly, because trained immunity operates independently of antigen recognition, it offers an advantage over adaptive immunity in countering rapidly evolving RNA viruses.

Nevertheless, memory‐like reprogramming is not uniformly protective. Depending on the intensity and nature of the initial stimulus, macrophages may adopt either a trained state with heightened responsiveness or a tolerant state with impaired effector activity. Low‐dose LPS or β‐glucan exposure establishes latent enhancers (H3K4me1) that promote robust cytokine responses upon restimulation, whereas high‐dose LPS or severe systemic inflammation induces repressive histone marks (H3K9me2, H3K27me2), suppressing cytokine production and phagocytosis.^[^ ^172^, ^173^ ^]^ In the lung, this can manifest as long‐term immunoparalysis of AMs, with impaired bacterial clearance persisting weeks after pneumonia or sepsis.^[^ ^172^ ^]^ Clinically, tolerogenic memory increases susceptibility to hospital‐acquired pneumonia, and such states can persist in human monocytes and AMs for months following systemic inflammation.^[^ ^172^ ^]^

Recent work further highlights the importance of epigenetic remodeling in shaping macrophage memory within the lung microenvironment. In a muco‐obstructive airway model, AMs exhibited widespread DNA hypomethylation and increased chromatin accessibility at promoter and enhancer regions, accompanied by enrichment of IRF, NF‐κB, C/EBP, STAT6, and ATF3 motifs. These epigenetic changes were linked to a mixed activation state, impaired efferocytosis, and exaggerated responses to secondary stimuli. Notably, exposing wild‐type macrophages to native mucus could reproduce similar transcriptional and functional alterations, demonstrating that local tissue cues can impose durable epigenetic reprogramming independent of infection.^[^ ^174^ ^]^

Collectively, these findings underscore the dual nature of macrophage memory. On one hand, trained Memory‐Ms provide broad, antigen‐independent protection that complements adaptive immunity; on the other, tolerogenic states compromise host defense and predispose individuals recovering from severe illness to secondary infections. Modulating macrophage memory—by enhancing beneficial training or reversing tolerance might offer therapeutic strategy.^[^ ^172^, ^175^, ^176^ ^]^ But the differentiation, persistence, and tissue distribution of Memory‐Ms remain poorly defined. Key questions include how acute infection, systemic vaccination, or microbial metabolites shape distinct memory trajectories, and how T cell–dependent and T cell–independent pathways converge in vivo. Addressing these gaps will be crucial for translating macrophage memory into clinical interventions for pulmonary infections.

### 3.6 Senescent Macrophages

Senescent macrophages (Senesc‐Ms) constitute a distinct subpopulation that accumulates with age and chronic inflammatory stress. Senesc‐Ms exhibit profound phenotypic and functional changes, including dysregulated gene expression, increased transcriptional noise, reduced proliferative capacity, and altered cytokine profiles. The progressive decline in macrophage function is a hallmark of aging‐associated chronic inflammation.^[^ ^177^ ^]^

In the aging lung, embryonically‐derived tissue‐resident AMs gradually lose their proliferative ability and are increasingly replaced by monocyte‐derived AMs.^[^ ^178^ ^]^ This shift in origin is accelerated by repeated inflammatory insults such as respiratory infections. As a result, senescent AMs not only emerge in aged individuals but are also observed in young individuals recovering from severe or recurrent infections, indicating a broader relevance of macrophage senescence beyond chronological aging.^[^ ^179^ ^]^

Functionally, Senesc‐AMs exhibit impaired pathogen clearance and compromised tissue homeostasis, contributing to increased susceptibility to infections and the development of chronic inflammatory lung diseases. Interestingly, while the expression of MHC II molecules increases in Senesc‐AMs, which are primarily driven by IFN‐γ signaling from T cells during inflammation, this does not fully compensate for their decreasing numbers and diminished functional capacity.^[^ ^169^, ^178^, ^180^ ^]^ Senesc‐AMs also exhibit a distinctive proinflammatory transcriptional profile. Compared to their younger counterparts, they display elevated mRNA levels of cytokines and chemokines, including *CCL2*, *IFN‐β*, *IL‐10*, *IL‐12p40*, *TNF‐α*, and *MIF*. These molecules are enriched in alveolar lining fluid, contributing to a chronic inflammatory microenvironment that impairs immune regulation and promotes tissue damage.^[^ ^181^ ^]^

Aging not only alters macrophage function but also reshapes their ontogeny. In aged lungs, monocyte‐derived AMs increasingly populate the alveolar niche, which is marked by *Siglec‐F* ^lo^, *MHC II* ^hi^, and *CCR2* ^hi^ expression, along with monocytic markers such as *Ly6C*, *CX_3_CR1*, and *CD115*.^[^ ^178^, ^181^, ^182^, ^183^, ^184^ ^]^ This phenotypic switch is further influenced by the aged pulmonary microenvironment.

Importantly, senescence‐like features are not exclusive to aging but can be induced by acute infections. During sublethal *Streptococcus pneumoniae* (*S.P*.) infection, embryonically‐derived tissue‐resident AMs survive the initial insult but exhibit senescent phenotypes—reduced self‐renewal, elevated senescence‐associated β‐galactosidase (SA‐β‐Gal) activity, and transcriptional signatures associated with metabolic decline.^[^ ^185^ ^]^ These Senesc‐AMs are gradually outcompeted by monocyte‐derived AMs within 2–3 weeks post‐infection. Transitional macrophages (Trans‐Macs), identified during this recovery phase, exhibit a phenotypic continuum from CD11b^hi^CD11c^−^Siglec‐F^−^ monocyte‐like cells to mature CD11b^−^CD11c^+^Siglec‐F^+^ AMs.^[^ ^179^, ^186^ ^]^ Bone marrow chimera experiments and adoptive transfer assays confirm that Trans‐Macs represent a differentiation intermediate between recruited monocytes and mature monocyte‐derived AMs. In contrast, Senesc‐AMs show diminished competitive fitness in vivo, suggesting that bacterial pneumonia drives a functional decline in embryonic AMs, ultimately favoring their replacement by monocyte‐derived populations.^[^ ^179^ ^]^

Several advanced tools are now available to characterize Senesc‐Ms through various hallmarks, including cell cycle arrest, the expression of cyclin‐dependent kinase (CDK) inhibitor proteins (e.g., *p16^Ink4a^* and *p21^CIP1^*), senescence‐associated β‐galactosidase (SA‐β‐Gal) activity, and the senescence‐associated secretory phenotype (SASP).^[^ ^187^, ^188^, ^189^ ^]^ Lineage‐tracing techniques reveal that *p16^Ink4a+^* Senesc‐Ms exhibit reduced vascular endothelial growth factor (VEGF) production, which may contribute to the pathogenesis of fibrotic pulmonary diseases.^[^ ^189^, ^190^ ^]^ Nevertheless, distinguishing true senescence from other dysfunctional states remains a major challenge. *p16^Ink4a^* and SA‐β‐Gal are widely used as canonical markers of senescent cells, and clearance of *p16^Ink4a^* ^+^ cells has been shown to improve healthspan in aged mice.^[^ ^191^ ^]^ However, recent findings raise concerns about the specificity of these markers in macrophages. *p16^Ink4a^*/SA‐β‐Gal–positive macrophages have been detected in the adipose tissue of aged mice and in the peritoneal cavity of young animals after injection of alginate‐encapsulated senescent cells, suggesting that these markers may not be exclusive to bona fide senescent cells. Moreover, unlike the classical p53‐dependent pathway that underlies senescence induction in most cell types, macrophages can express *p16^Ink4a^* and SA‐β‐Gal in a p53‐independent manner, as demonstrated in p53‐null mice. Their expression is also dynamically regulated by microenvironmental cues: M2‐polarizing cytokines such as IL‐4 and IL‐13 enhance *p16^Ink4a^* promoter activity through JAK–STAT signaling, while poly(I:C) treatment significantly reduces *p16^Ink4a^* expression in alginate‐bead models and in aged mice without affecting macrophage viability. These observations highlight the context‐dependent and reversible nature of *p16^Ink4a^*/SA‐β‐Gal expression in macrophages, underscoring the need to discriminate true senescence from transient activation or differentiation states.^[^ ^192^ ^]^

## 4 Conclusion and Future Perspectives

In this review, we propose an integrative classification system for identifying macrophage subtypes in pulmonary infections on the basis of single‐cell omics and functional phenotypic characteristics. This framework provides a more precise understanding of macrophage function and phenotype transitions during infection, highlighting key macrophage populations as potential therapeutic targets.

Macrophages are highly plastic and continuously adapt to changes in the microenvironment during infection.^[^ ^154^ ^]^ The macrophage subtypes discussed in this review are defined by subtle differences in their transcriptional profiles and functional characteristics, which are continuously shaped by pathogen interactions, the immune microenvironment, and other immune cells.^[^ ^6^ ^]^ Importantly, these macrophage subsets are not strictly mutually exclusive but instead represent preferential functional states along a continuous spectrum of activation. In this sense, the six‐category model should not be interpreted as rigidly discrete groups, but rather as conceptual “nodes” on a continuum of macrophage plasticity, where distinct functional biases become sufficiently robust to warrant classification as subpopulations. Moreover, molecular features, pathways, and metabolic changes can be highly pathogen specific, with different pathogens inducing distinct alterations.^[^ ^40^, ^41^ ^]^ Macrophages within the same functional subset may also originate from different sources, including tissue‐resident macrophages undergoing reprogramming or monocyte‐derived macrophages recruited from the peripheral circulation during infection.^[^ ^7^, ^160^ ^]^ Several of the functional subtypes are enriched in, or preferentially associated with, particular anatomical niches. For example, *Nos2* ^+^ Inflam‐Ms are predominantly monocyte‐derived and often localize to the interstitial compartment,^[^ ^106^ ^]^ whereas a subset of *SPP1* ^hi^ Reg‐Ms is enriched in the alveolar space.^[^ ^15^ ^]^ Similarly, Prolif‐Ms have been described both in self‐renewing AM pools and in replenished IM compartments.^[^ ^9^, ^160^, ^161^ ^]^ In addition, senescent macrophages frequently emerge within the alveolar niche as aging or recurrent infection progressively impairs the embryonic AM pool.^[^ ^179^ ^]^

Macrophage identity and function are profoundly influenced by interactions with surrounding cell types. Pluripotent stem cell–derived co‐culture models have demonstrated that differentially polarized macrophages exert opposing effects on viral replication, epithelial survival, and inflammatory output, underscoring the decisive role of macrophage–epithelial crosstalk in shaping infection outcomes.^[^ ^193^ ^]^ Likewise, iPSC‐derived mesenchymal stromal cells have been shown to dampen macrophage and neutrophil infiltration, reduce oxidative stress, and promote epithelial repair through paracrine factors as well as contact‐dependent mechanisms.^[^ ^194^ ^]^ These findings highlight that macrophages not only act as independent immune modulators, but also connect multicellular networks that orchestrate immunity, tissue remodeling, and repair.

It is critical to recognize functionally distinct macrophage subgroups, which then can serve as diagnostic or therapeutic targets. Given the continuous spectrum of macrophage plasticity, future studies employing single‐cell omics are expected to uncover additional, previously unrecognized macrophage subtypes.^[^ ^15^ ^]^ While these technological advances offer increasingly refined resolution, a critical challenge lies in balancing this granularity with conceptual clarity. Future progress depends on developing standardized single‐cell analysis pipelines, which can provide more consistent and accurate insights into macrophage functions during infection.

Despite significant advances in understanding macrophage heterogeneity through single‐cell technologies, several limitations remain. Current scRNA‐seq methods may underrepresent rare cell populations and capture only a snapshot of dynamic cellular processes. Furthermore, variability in sample preparation, sequencing depth, and bioinformatic analysis pipelines can introduce biases, affecting reproducibility across different studies.^[^ ^195^ ^]^ In addition, technical challenges remain in single‐cell omics approaches, including biases in cell capture, limited spatial resolution, and difficulties in correlating transcriptional profiles with functional outcomes. This might have the risk of oversimplifying the highly dynamic and transitional states of macrophages during infection. Emerging approaches such as RNA velocity and pseudotime analysis might be applied to infer dynamic state transitions of macrophages. Also, combining scRNA‐seq with spatial transcriptomics or spatial proteomics enables the localization of macrophage subsets within the pulmonary microenvironment, thereby linking transcriptional states to tissue architecture. Furthermore, experimental strategies such as lineage tracing and multi‐omics integration (e.g., scATAC‐seq, metabolomics) could enhance the ability to resolve rare subsets and uncover regulatory mechanisms. Future research should focus on integrating multi‐omics data, incorporating spatial information, and developing standardized protocols to address these limitations and gain a more comprehensive understanding of macrophage heterogeneity in pulmonary infections.

In conclusion, exploring the relationships between newly identified macrophage subtypes and specific infection types, as well as different stages of infection, could reveal molecular signatures with diagnostic, prognostic, and therapeutic potential. Advanced technologies such as spatial transcriptomics, lineage tracing, and stereo‐seq are essential for elucidating macrophage differentiation and functional shifts during infection, enabling the identification of key signaling pathways and microenvironmental cues that drive these processes.^[^ ^16^, ^110^ ^]^ Targeting macrophage subtypes based on their molecular signatures holds great promise for developing personalized therapies that can prevent tissue damage while optimizing the immune response in pulmonary infections. Moreover, incorporating macrophage‐targeting strategies into vaccine development could offer a novel approach to combating global pandemics, such as the ongoing SARS‐CoV‐2 crisis.^[^ ^175^, ^176^ ^]^ Nevertheless, translating these approaches into clinical practice faces substantial challenges. Safety and specificity remain critical concerns, as off‐target effects or unintended immune activation could compromise host homeostasis. Delivery to pulmonary macrophages is particularly difficult due to biological barriers, and the durability and long‐term consequences of macrophage reprogramming remain uncertain. In addition, the high cost and complex manufacturing of cell‐based therapies pose barriers to large‐scale application. Addressing these issues will be essential to realize the therapeutic potential of macrophage‐based interventions and their impact on global infectious disease control.

## Conflict of Interest

The authors declare no conflict of interest.

## Author Contributions

W.H.Y. supervised the work and revised the manuscript. L.Z.H. led the conceptualization, drafted the manuscript, and created all figures. Z.Y.X. improved and refined the manuscript. Z.Y. prepared Table 1 and assisted with outline refinement. L.Z.H., Z.Y.X., and Z.Y. contributed equally to this work. All the authors approved the final manuscript.
